# Microbiome-mediated pharmacology of ginseng: Mechanistic insights into metabolic regulation and therapeutic potential

**DOI:** 10.1016/j.jgr.2026.101023

**Published:** 2026-03-24

**Authors:** Woo Kyu Kang, Sun-Young Hwang, Hyunjin Kang, Jin Won Hyun, Sang-Kyu Kim, Mee-Hyun Lee

**Affiliations:** a Department of Neurobiology, University of Massachusetts Chan Medical School, Worcester, MA, 01605, USA; b College of Korean Medicine, Dongshin University, Naju, 58245, Republic of Korea; c Department of Biochemistry, College of Medicine and Jeju Natural Medicine Research Center, Jeju National University, Jeju, 63243, Republic of Korea; d R&D Headquarters, Korea Ginseng Corporation, 456, Galhyeon-dong, Gwacheon-si, Gyeonggi-do, 13840, Republic of Korea; e Korean Medicine Convergence Research Institute, College of Korean Medicine, Dongshin University, Naju, 58245, Republic of Korea

**Keywords:** Ginseng, Gut microbiota, Probiotics, Ginseng–microbiota interactions, Microbiome-informed ginseng therapies

## Abstract

Ginseng, a traditional medicinal herb with a favorable safety profile, has long been used to promote systemic health. Recent studies reveal that many of its beneficial effects are mediated through interactions with the gut microbiota. Microbial enzymes convert parent ginsenosides into more absorbable and bioactive metabolites such as compound K, while ginseng reciprocally remodels the microbial community and metabolite composition by promoting the growth of beneficial taxa including *Akkermansia*, *Bifidobacterium*, and *Lactobacillus*. These bidirectional interactions modulate host metabolic, immune, and intestinal barrier functions. The ginseng–microbiome interplay regulates microbial and host-derived metabolites such as short-chain fatty acids, bile acids, and indole derivatives, which in turn activate key signaling pathways including FXR/TGR5, FFAR, AMPK, and Nrf2. Through these mechanisms, ginseng improves lipid metabolism, enhances insulin sensitivity, alleviates low-grade inflammation, and ameliorates metabolic abnormalities such as obesity, insulin resistance, and nonalcoholic fatty liver disease (NAFLD). This review provides a comprehensive synthesis of the ginseng–microbiota metabolic axis, focusing on its mechanistic basis in metabolic regulation and related disorders. We also highlight the therapeutic convergence between ginseng and probiotics possessing ginsenoside-hydrolyzing enzymes, discuss strategies for strain selection and co-administration, and outline future directions in precision, microbiome-informed formulations and clinical trial design. Collectively, current evidence supports the ginseng–microbiota interactions as a promising therapeutic platform for restoring metabolic homeostasis and managing metabolic diseases.

## Graphical abstract

**Figure:**
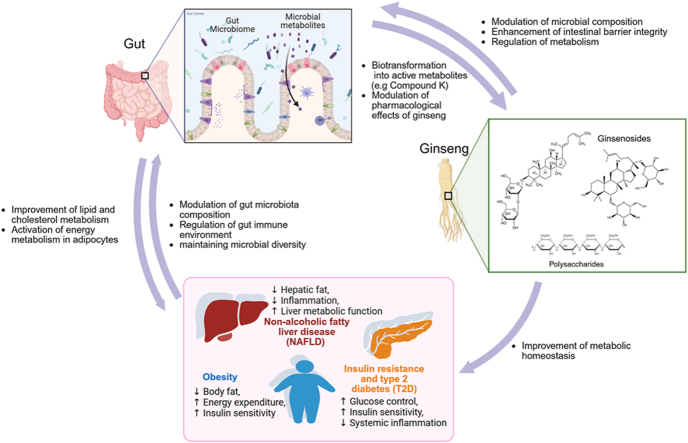

## 1 Introduction

The human gut and its resident microbiota form a complex and dynamic ecosystem that is central to metabolic homeostasis. This diverse community—comprising bacteria, archaea, viruses, and eukaryotic microorganisms—supports key physiological functions such as nutrient harvest, energy metabolism, and maintenance of intestinal barrier integrity, which underpins host metabolism [1]. Disruption of this finely balanced ecosystem, known as dysbiosis, is strongly linked to metabolic disorders such as obesity, insulin resistance, type 2 diabetes (T2D), and non-alcoholic fatty liver disease (NAFLD) [2,3].

A key concept in microbiome research is the “gut–organ axis,” which refers to bidirectional communication pathways between the gut and distant organs via microbial metabolites (e.g., short‐chain fatty acids (SCFAs) and bile acids), endocrine and immune signaling pathways [[3], [4], [5]]. Through these pathways, particularly via metabolite signaling, the gut microbiota influences not only local functions such as maintaining the intestinal barrier but also metabolic regulation in distant organs including the liver and adipose tissue. For example, microbial metabolites regulate hepatic lipid metabolism and adipose inflammation, while bile acid signaling and incretin modulation link gut microbial activity to glucose and lipid homeostasis [4,5]. Given this extensive systemic influence, the gut microbiota serves both as a sensitive biomarker of metabolic risk and a promising therapeutic target [[1], [2], [3], [4], [5]].

Korean ginseng (*Panax ginseng* Meyer) has been used for centuries in traditional East Asian medicine, particularly in Korea, to enhance vitality and promote metabolic resilience [6]. Among the various ginseng preparations, Korean red ginseng—produced through a steaming and drying process—represents a widely used and relatively standardized processed form of *Panax ginseng* [6,7]. This steaming process not only improves shelf stability but also reshapes the saponin profile, enriching less-polar, so-called “rare” ginsenosides such as Rg3, Rg5, and Rk1, which have frequently been reported to exhibit stronger biological activities than their parent compounds [[7], [8], [9]].

In clinical contexts, standardized *Panax ginseng* preparations have been evaluated as adjunct interventions with acceptable tolerability across diverse populations, including metabolically vulnerable groups, supporting the translational feasibility of ginseng as a systemic botanical therapeutic [6,10]. Beyond metabolic regulation, ginseng and its bioactive constituents have been reported to influence multiple physiological domains, including immune homeostasis, inflammatory responses, and oxidative stress modulation, which are increasingly recognized as metabolically relevant processes [9,11,12]. Concurrently, accumulating mechanistic evidence indicates that ginseng-derived compounds are associated with organ-specific metabolic reprogramming, including improvements in mitochondrial function and energy metabolism in peripheral tissues [7,11,13]. Such broad pharmacological effects are difficult to fully explain by direct, organ-specific actions alone and can be more coherently interpreted through the gut–liver–metabolic axis, in which microbial biotransformation and metabolite signaling integrate diverse systemic outcomes [14,15]. Notably, a substantial proportion of these systemic effects converge on pathways regulating energy metabolism, oxidative stress, and chronic low-grade inflammation—core processes underlying the pathophysiology of obesity, NAFLD, and T2D [1].

Building on this metabolic framework, ginseng, derived from *Panax* species such as *P. ginseng*, *P. quinquefolius*, and *P. notoginseng* exerts its pharmacological effects through bidirectional interactions with the gut microbiota, including modulation of microbial composition and microbiota-mediated biotransformation of ginsenosides into more bioactive metabolites [16]. Contemporary research indicates that many of ginseng's metabolic therapeutic effects are mediated, at least in part, through interactions with the gut microbiota, while gut microbiota–dependent biotransformation critically shapes the pharmacokinetics and systemic exposure of ginsenosides [14,15,17]. These interactions operate bidirectionally. Intestinal microbes transform parent ginsenosides—such as Rb1, Rc, and Re—into more absorbable and pharmacologically active metabolites (e.g., compound K, F2, and Rh1) through deglycosylation and related reactions, thereby altering intestinal uptake and systemic exposure [[18], [19], [20], [21]]. Conversely, ginseng components, including saponins and polysaccharides, remodel community structure and function by enriching beneficial taxa such as *Bifidobacterium*, *Lactobacillus*, and *Akkermansia*, while modulating microbial metabolite profiles that are tightly linked to host energy and glucose metabolism [15,22,23]. This reciprocal relationship provides a mechanistic basis for ginseng's broad efficacy within the gut–liver–metabolic axis [14,16]. Importantly, studies using fecal microbiota transplantation (FMT) and germ-free animal models have shown that transferring gut microbes from ginseng-treated animals is sufficient to reproduce its metabolic benefits such as improved lipid metabolism and insulin sensitivity, thereby establishing a causal role for the gut microbiota in mediating ginseng's systemic effects [15,24].

Accumulating evidence suggests that these microbial functions are critical determinants of metabolic health. Mechanistically, microbially derived metabolites such as SCFAs and bile acids act as major mediators linking the gut microbiota to host metabolic regulation [4,5]. Special attention has been given to SCFA and bile acid signaling, farnesoid X receptor (FXR)/G protein–coupled bile acid receptor 1 (TGR5) modulation, and ginsenoside biotransformation as mechanistic links between microbial activity and host metabolism [4,15,[18], [19], [20], [21]]. Ginseng intake has been shown to promote SCFA-producing and bile acid–modifying microbes, thereby enhancing metabolic signaling along the gut–liver axis and alleviating steatosis and insulin resistance [[25], [26], [27], [28]].

In this review, we focus specifically on the gut–liver–metabolic axis to synthesize evidence on how ginseng and the gut microbiota cooperatively regulate obesity, NAFLD, and T2D. We explore how ginseng and its major bioactive components influence, and are influenced by, the gut microbiome, drawing on findings from *in vitro*, animal, and human studies. We also highlight emerging tools such as germ-free models, metagenome-assembled genomes, and multi-omics technologies that are advancing mechanistic insights into ginseng–microbiota interactions. Finally, we discuss the translational implications of these findings, with a focus on the potential for microbiome-informed metabolic interventions, personalized probiotic combinations, and standardized ginseng formulations.

## 2 Bioactive components of ginseng and microbiome interactions

**Fig. 1:**
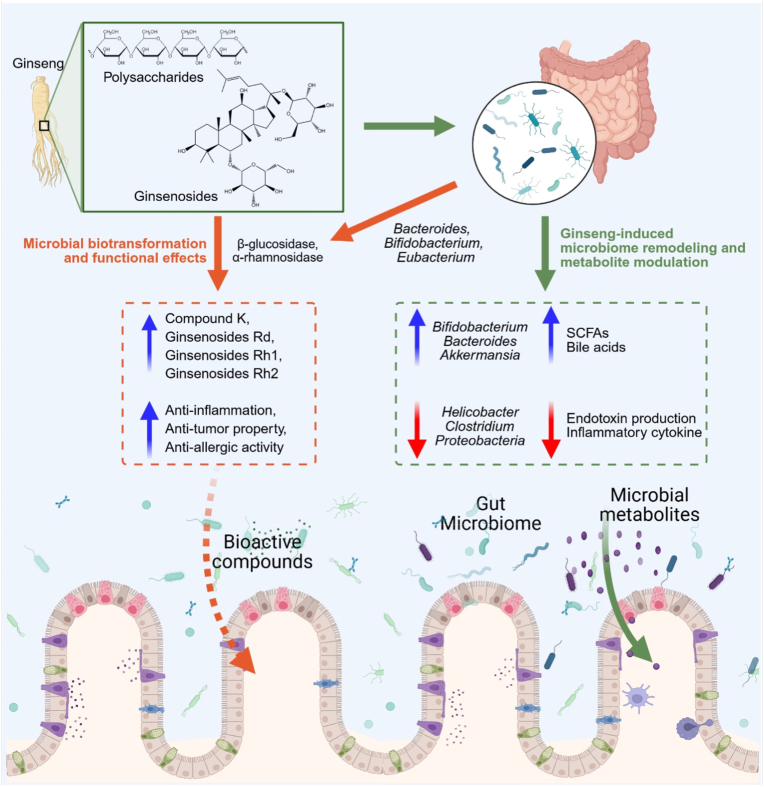
**Bidirectional interactions between ginseng bioactives and the gut microbiota.** Gut microbial enzymes convert ginsenosides into bioactive metabolites with enhanced intestinal absorption and pharmacological potency. In turn, ginseng reshapes the gut microbiota, enriching beneficial taxa and promoting the production of health-promoting metabolites, while suppressing pro-inflammatory or pathogenic genera. Ginseng polysaccharides (GPs) function as prebiotics, fostering beneficial microbial growth and stimulating the synthesis of short-chain fatty acids (SCFAs) and secondary bile acids. These microbial and metabolic shifts amplify ginsenoside metabolism and SCFAs production, establishing a reinforcing feedback loop. Together, these reciprocal interactions form a synergistic axis underlying ginseng's systemic health benefits.

The systemic metabolic benefits of ginseng are increasingly recognized as the result of complex, bidirectional interactions between its bioactive components and the gut microbiota. Major constituents, such as ginsenosides and polysaccharides, not only serve as substrates for microbial metabolism but also actively remodel the composition and function of the gut microbial community. Notably, gut microbes including *Bacteroides*, *Bifidobacterium*, and *Eubacterium* produce glycosidase enzymes that deglycosylate ginsenosides into more bioactive metabolites such as compound K. Conversely, ginseng supplementation enriches beneficial bacteria such as *Bifidobacterium*, *Bacteroides*, and *Akkermansia*, while suppressing potentially harmful taxa. This synergistic relationship between ginseng and the gut microbiota constitutes a critical axis underpinning its pharmacological activities and the metabolic health benefits (Fig. 1) [15].

a Ginsenosides: microbial biotransformation and functional effects

Ginsenosides, the primary bioactive compounds in ginseng, are triterpene saponins classified into protopanaxadiol (PPD) and protopanaxatriol (PPT) types based on their aglycone structure and sugar moieties [11]. Although these compounds have potent pharmacological potential, their native forms such as Rb1, Rc, Rd (PPD-type), and Rg1, Re (PPT-type) exhibit limited intestinal absorption due to their glycosylated, water-soluble structure [11]. Upon reaching the colon, ginsenosides undergo sequential deglycosylation by gut microbial enzymes, particularly β-glucosidase and α-rhamnosidase, yielding more lipophilic and absorbable metabolites [18,21]. Genera such as *Bacteroides*, *Bifidobacterium*, *Eubacterium*, and *Prevotella* are key contributors to this transformation process [17]. Certain fungal species and plant endophytes can also facilitate deglycosylation, although their overall contribution is generally less dominant than that of gut bacteria [19,20].

Microbial deglycosylation of ginsenosides markedly reduces polarity and enhances lipophilicity, facilitating greater intestinal absorption and systemic bioavailability. The resulting metabolites such as compound K, Rh1, and F2 exhibit enhanced metabolic activity, such as improvement of lipid metabolism, attenuation of hepatic steatosis, and enhancement of insulin sensitivity. For example, ginsenoside Rb1 is sequentially deglycosylated into intermediates such as Rd and F2 and ultimately compound K, a metabolite known to ameliorate hepatic lipid accumulation and glucose intolerance [17]. Similarly, Rg1 and Re are converted into Rh1 and Rh2, which display regulatory effects on adipogenesis and inflammatory signaling relevant to metabolic disorders [18].

The multistep nature of this microbial transformation can generate a spectrum of metabolites with distinct and often superior metabolic bioactivities. Differences in microbiota composition, particularly in the abundance and enzymatic activity of glycosidase-producing taxa, lead to person-to-person differences in pharmacokinetics and metabolic efficacy [21]. Thus, the gut microbiome serves as a critical determinant of ginseng bioavailability and metabolic outcomes.

b Ginseng-induced microbiome remodeling and metabolite modulation

Beyond serving as substrates for microbial metabolism, ginseng and its constituents actively reshape the gut microbiota. Supplementation with ginseng has been consistently associated with increased abundance of beneficial taxa such as *Lactobacillus*, *Bifidobacterium*, *Akkermansia*, and *Bacteroides*, which contribute to gut barrier integrity and the production of SCFAs including butyrate, acetate, and propionate [16]. These SCFAs act as key metabolic regulators, promoting lipid oxidation, improving insulin sensitivity, and maintaining energy balance. In contrast, potentially pathogenic or pro-inflammatory genera such as *Helicobacter*, *Clostridium*, and *Proteobacteria* are suppressed, leading to reduced endotoxin production and mitigation of metabolic inflammation [16].

In addition to broad taxonomic effects, certain ginsenosides may exert targeted effects on microbial composition. For example, ginsenosides Rk3 has been shown to selectively enrich *Blautia* spp., known for SCFAs production and anti-inflammatory properties [29]. These targeted microbial shifts underscore the capacity of individual ginsenosides to fine-tune the gut ecosystem in ways that improve metabolic outcomes.

Importantly, ginseng-induced remodeling extends to microbial functional capacity. The enriched microbial community exhibits increased potential to metabolize dietary lipids and carbohydrates and to produce SCFAs and bile acid intermediates that activate host nuclear receptors such as the FXR and TGR5. Activation of these receptors promotes bile acid turnover, lipid catabolism, and thermogenic energy expenditure, while suppressing hepatic lipogenesis and inflammation. These coordinated changes establish a mechanistic basis for ginseng's actions in improving lipid and glucose metabolism and preventing metabolic disorders such as obesity, NAFLD, and T2D**.**

c Polysaccharides: prebiotic effects and immune modulation

Ginseng polysaccharides (GPs), particularly those in water-soluble acidic and neutral fractions, are another major group of bioactive compounds with significant prebiotic potential. These complex carbohydrates, including pectin-type polysaccharides, starch-like glucans, and arabinogalactans, are resistant to human digestive enzymes and reach the colon intact, where they are fermented by the gut microbiota into SCFAs such as acetate, propionate, and butyrate [22].

GPs promote the growth of beneficial bacteria such as *Lactobacillus*, *Bifidobacterium*, and *Akkermansia*, thereby enhancing SCFA production and facilitating ginsenoside metabolism [22]. Co‐administration of GPs and ginsenosides has been shown to modulate gut microbial metabolism, restore the *Firmicutes*/*Bacteroidetes* ratio, reduce metabolic endotoxemia, and improve energy homeostasis [23].

Collectively, these findings emphasize that ginseng's metabolic efficacy arises not only from its native compounds but also from the dynamic metabolic outputs generated through its interaction with the gut microbiota.

## 3 Metabolic regulation by ginseng through microbiota-dependent mechanisms

Ginseng was traditionally believed to exert its therapeutic effects through the direct action of its bioactive compounds on host organs. However, this mechanism alone does not fully account for the broad range of metabolic benefits associated with ginseng.

Recent evidence demonstrates that many of ginseng's effects are mediated through complex and dynamic interactions with the gut microbiota. These interactions involve selective modulation of microbial community composition and enhancement of key microbial metabolites, including SCFAs and secondary bile acids, which serve as central mediators linking gut microbial activity to host lipid and glucose metabolism via the gut–liver axis. SCFAs and bile acid derivatives engage host receptors such as GPR41/43, FXR, and TGR5, thereby coordinating lipid oxidation, glucose utilization, mitochondrial efficiency, and energy expenditure, which collectively establish a biochemical foundation for ginseng's metabolic actions [4,5,15,[25], [26], [27]].

Ginseng's influence on the gut microbiota is particularly evident in metabolic disorders, including obesity, NAFLD, and T2D, where the gut–liver–metabolic axis integrates microbial and host signaling to regulate hepatic function, insulin sensitivity, and systemic energy regulation [15,[25], [26], [27], [28]]. Among these conditions, obesity, NAFLD, and T2D have been most extensively investigated as microbiota-dependent targets of ginseng action.

This section summarizes how ginseng remodels the gut microbial ecosystem under metabolic stress conditions, providing a mechanistic framework for understanding its microbiota-mediated therapeutic potential (Table 1).

a Obesity

**Table 1:** Systemic health benefits of ginseng mediated by microbiota-dependent mechanisms.

Condition	Key Effects	Microbiota-Mediated Mechanisms	Reference
Obesity	Reduces body weight, fat mass, improves glucose balance & insulin sensitivity	↑*Akkermansia*, *Verrucomicrobia*; ↓*Firmicutes*; boosts SCFAs; regulates bile acids; activates leptin–AMPK/STAT3 & FFAR4 pathways; promotes thermogenesis	[23,24,[28], [29], [30]]
NAFLD	Reduces liver fat, improves lipid metabolism, protects against inflammation & oxidative stress	↑*Akkermansia*, *Bacteroides*; ↓*Firmicutes*/*Bacteroidetes* ratio; boosts SCFAs; alters bile acid metabolism; activates LKB1–AMPK–mTOR & FXR signaling; reduces NF-κB	[25,26,[31], [32], [33], [34], [35], [36]]
T2D	Improves glucose control, enhances insulin sensitivity, lowers inflammation	↑*Akkermansia*, *Lactobacillus*, *Bifidobacterium*; enhances GLP-1; activates AMPK/PI3K/AKT & TGR5; regulates ferroptosis via Nrf2; lowers TNF-α & IL-6	[[37], [38], [39], [40], [41], [42], [43], [44]]

SCFAs, short-chain fatty acids; AMPK, AMP-activated protein kinase; STAT3, signal transducer and activator of transcription 3; FFAR4, free fatty acid receptor 4; NAFLD, non-alcoholic fatty liver disease; LKB1, liver kinase B1; mTOR, mechanistic target of rapamycin; FXR, farnesoid X receptor; NF-κB, nuclear factor kappa B; T2D, type 2 diabetes; GLP-1, glucagon-like peptide-1; PI3K, phosphoinositide 3-kinase; AKT, protein kinase B; TGR5, G protein–coupled bile acid receptor 1; Nrf2, nuclear factor erythroid 2–related factor 2; TNF-α, tumor necrosis factor-alpha; IL-6, interleukin-6.

Obesity is a major metabolic disorder characterized by energy imbalance, chronic low-grade inflammation, and altered lipid metabolism, in which the gut microbiota plays a central regulatory role in energy homeostasis and adipose function. The intricate relationship between obesity and gut microbiota dysbiosis has emerged as a crucial therapeutic target in metabolic disease management. Emerging evidence demonstrates that ginseng and its bioactive compounds modulate obesity primarily through gut microbiota remodeling and reprogramming of host metabolic signaling. In diet-induced obesity models, supplementation with ginseng significantly reduces body weight gain and adipose accumulation while improving glucose tolerance and insulin sensitivity. These effects coincide with increased abundance of beneficial taxa, particularly *Akkermansia*, *Proteobacteria*, and *Verrucomicrobia*, alongside a reduction in obesity-associated phyla such as *Firmicutes* and *Tenericutes,* indicating the restoration of microbial homeostasis within the gut–liver metabolic axis [30].

Mechanistically, several pathways contribute to these effects. The microbiota–fatty acid axis is a primary mechanism: ginsenoside Rb1 promotes long-chain fatty acid production and activates the free fatty acid receptor 4 (FFAR4) signaling in the colon, thereby promoting insulin sensitivity and lipid utilization [30]. Specific microbial metabolites also play key roles; for instance, *Enterococcus faecalis*–derived myristoleic acid activates brown adipose tissue thermogenesis and reduces fat accumulation [31]. Additionally, ginsenoside Rg1 modulates bile acid metabolism via microbiota alterations, thereby modulating bile salt hydrolase–producing taxa, leading to enhanced TGR5 activation and hepatic lipid turnover [25].

*Panax notoginseng* saponins also stimulate beige adipocyte formation and thermogenesis via a microbiota-dependent leptin– AMP-activated protein kinase (AMPK)/signal transducer and activator of transcription 3 (STAT3) signaling pathway, confirming the integral role of gut microbes in mediating ginseng's metabolic benefits [26].

Human trials, though still limited, provide consistent evidence. Ginseng supplementation in obese individuals leads to significant reductions in body mass index, waist circumference, and fasting glucose, with response magnitude correlating with baseline microbiota composition [32].

Together, these findings establish ginseng as a potent microbiota-directed metabolic modulator that improves energy balance and insulin sensitivity through coordinated regulation of microbial composition, bile acid metabolism, and thermogenic signaling.

b NAFLD

NAFLD, defined by hepatic lipid accumulation, is closely associated with gut microbiota dysbiosis and impaired bile acid signaling. Recent evidence supports that ginseng, particularly ginsenosides Rg5, Re, Rd, and Rh4, exerts hepatoprotective effects by modulating both liver and microbiota function. In high-fat diet-induced NAFLD models, ginseng compounds reduce hepatic lipid accumulation via suppression of lipogenic gene expression (e.g., SREBP-1c, FAS, ACC-1), upregulation of lipolytic pathways (CPT-1a), and activation of liver kinase B1 (LKB1)–AMPK– mechanistic target of rapamycin (mTOR) signaling [27,28].

Concomitantly, ginseng-induced remodeling of gut microbiota enriches beneficial taxa such as *Akkermansia*, *Lactobacillus*, and *Bacteroides*, restoring the *Firmicutes*/*Bacteroidetes* ratio and improving intestinal barrier integrity [14,15,30,32]. These microbial changes enhance SCFA production and shift bile acid profiles toward increased levels of secondary bile acids that activate FXR and TGR5 signaling [1,4,28,33,34]. FXR activation in the liver and intestine suppresses hepatic de novo lipogenesis, while TGR5 activation in brown adipose tissue stimulates energy expenditure and thermogenesis, providing a systemic metabolic benefit [5,28,34].

Ginseng also exhibits potent anti-inflammatory effects through inhibition of nuclear factor kappa B (NF-κB) and phosphoinositide 3-kinase (PI3K)/protein kinase B (AKT) pathways [35]. Recent studies have revealed protection against oxidative stress through nuclear factor erythroid 2–related factor 2 (Nrf2)-dependent antioxidant responses and inhibition of ferroptosis via SLC7A11 and GPX4, further supporting its hepatoprotective effects [36]. Microbial metabolites such as butyrate and deoxycholic acid have been proposed as mediators of these effects, linking microbial activity to hepatic mitochondrial function and lipid oxidation [1,4,5].

The gut microbiota plays a central role in mediating these beneficial effects. Ginseng increases *Akkermansia*, *Bacteroides*, and *Lactobacillus*, and decreases the *Firmicutes/Bacteroidetes* ratio. These microbial shifts enhance SCFAs production and alter bile acid metabolism, improving intestinal barrier function [28,30]. The microbiota-dependent effects involve activation of FXR-dependent bile acid signaling, cholesterol metabolism, and AMPK pathway activation [33,37].

The microbiota dependency of these effects is confirmed through FMT studies: transplantation of gut microbes from ginseng-treated animals reproduces these hepatoprotective effects, while antibiotic-treated mice lose them [27,33,36]. Fermented and ginsenoside-enriched ginseng extracts show greater efficacy than crude extracts, likely due to improved microbial conversion efficiency [38]. Preliminary clinical studies report improvements in liver enzymes and favorable microbiota changes in NAFLD patients following ginseng treatment [39]. Collectively, these findings define ginseng as a modulator of the gut–liver–metabolic axis, and ginseng represents a promising microbiome-based therapeutic candidate for NAFLD.

c T2D

The interrelationship between insulin resistance, T2D, and gut microbiota is a growing focus in metabolic disease research. Ginseng and its active compounds, including ginsenosides Rb1, Rd, Rg1, Rg3, Rg5, and compound K, demonstrate potent glucose-lowering and insulin-sensitizing effects across multiple experimental models. These effects appear to be largely mediated through gut microbiota modulation, as evidenced by studies utilizing antibiotic depletion and fecal microbiota transplantation (FMT) approaches [36,40]. Notably, combination approaches using ginseng with other bioactive compounds have shown enhanced efficacy in improving insulin secretion and reducing insulin resistance while beneficially modulating the gut microbiome [41]. The mechanisms underlying these benefits operate through several interconnected pathways.

First, ginseng administration significantly alters gut microbiota composition, increasing SCFA-producing bacteria including *Lactobacillus*, *Bifidobacterium*, and *Roseburia*. Notably, treatment consistently enriches *Akkermansia muciniphila* populations, a bacterial species inversely correlated with insulin resistance [42].

Second, ginseng's metabolic benefits involve multiple signaling cascades. At the molecular level, ginseng enhances glucagon-like peptide-1 (GLP-1) secretion [34,40] and activates key metabolic pathways such as AMPK/PI3K/AKT pathway [43], and bile acid-TGR5 signaling [44], all of which improve insulin sensitivity and glucose homeostasis. Additionally, ferroptosis regulation via Nrf2 signaling has been identified as a novel mechanism underlying ginseng's metabolic benefits [36].

Third, ginseng also exerts potent anti-inflammatory effects in metabolic tissues. Treatment reduces pro-inflammatory cytokine levels (tumor necrosis factor-alpha (TNF-α), interleukin-6 (IL-6)) and gut-derived endotoxemia while improving intestinal barrier function [45]. Recent evidence suggests these anti-inflammatory effects are partially mediated through modulation of the autophagy-lysosome pathway [46], further contributing to improved glucose metabolism.

The translational potential of these findings is supported by emerging clinical evidence. Double-blind randomized controlled trials have demonstrated improved glycemic control and beneficial microbiota changes in patients with metabolic syndrome and T2D receiving ginseng supplementation [42]. These findings collectively establish a strong mechanistic foundation for ginseng's therapeutic potential in treating insulin resistance and T2D through microbiota-mediated pathways.

## 4 Therapeutic convergence and potential of ginseng and probiotics

The gut microbiota has emerged as a central therapeutic target in managing metabolic disorders. Disruptions in gut microbial balance, or dysbiosis, are strongly linked to metabolic disorders-including obesity, NAFLD, and T2D-where altered microbial metabolites directly impair glucose and lipid metabolism. This growing understanding has fueled the development of microbiome-based therapies, including conventional probiotics, engineered microbes, microbiota-targeted small molecules, and bacteriophage-based interventions. The regulatory approval of standardized microbiome products further underscores the clinical momentum toward precision microbiome interventions.

In parallel with these advances, ginseng has attracted significant interest as a microbiome-modulating metabolic agent. Accumulating evidence indicates that ginseng supplementation consistently enriches beneficial microbial taxa such as *Lactobacillus*, *Bifidobacterium*, and *Akkermansia*, while enhancing microbial production of bioactive metabolites such as SCFAs and modulating bile acid metabolism—two metabolite pathways central to lipid oxidation, glucose regulation, and intestinal barrier integrity [14,16]. These microbiota-mediated effects position ginseng as a promising metabolic intervention along the gut–liver axis.

A particularly intriguing aspect of ginseng–microbiota interactions is their mechanistic convergence with clinically validated probiotic strains used to treat metabolic diseases. Several *Lactobacillus plantarum* strains such as LMT1-48 [[47], [48], [49]], HAC01 [50,51], SKO-001 [52,53], and Q180 [54] demonstrate anti-obesity and lipid-lowering effects via modulation of adipogenesis and bile acid signaling, reflecting mechanisms observed in ginseng-treated models. *L. plantarum* TWK10 improves exercise performance and oxygen utilization, paralleling the ergogenic effects attributed to ginseng [55,56].

Similarly, *Bifidobacterium* strains exhibit effects consistent with those of ginseng. *B. breve* BR03 and B632 support pediatric gut and metabolic health [57], while *B. lactis* HN019 strengthens intestinal barrier function and reduces endotoxemia, resembling ginseng's ability to restore gut barrier integrity in models of metabolic dysfunction [58,59]. *B. breve* B-3 reduces adiposity and regulates bile acid metabolism, converging with ginseng's bile acid–FXR/TGR5–AMPK metabolic signaling axis [60,61].

Importantly, this convergence between ginseng and probiotics is bidirectional. While ginseng modulates the gut microbiota in a manner similar to that of probiotics, specific probiotic strains can further enhance the pharmacological activity of ginseng itself. Many ginsenosides require microbial transformation into more active deglycosylated metabolites, such as compound K, to exert systemic metabolic effects. Glycosidase enzymes produced by *Bifidobacterium*, *Lactobacillus*, and other commensals catalyze this conversion, and supplementation with strains expressing high levels of these enzymes can accelerate ginsenoside bioconversion and amplify metabolic efficacy [62].

This mechanistic convergence between ginseng and probiotics underscores a significant therapeutic opportunity. Combining ginseng with specific probiotic strains capable of both supporting gut microbial health and enhancing ginseng metabolism may yield synergistic interventions with maximized clinical efficacy. Such integrated strategies hold substantial translational promise for improving adiposity, hepatic lipid metabolism, insulin sensitivity, and overall metabolic homeostasis through coordinated modulation of microbial and host metabolic pathways.

## 5 Challenges and future directions

Despite promising preclinical findings and encouraging early clinical data, several challenges limit the full translation of ginseng–microbiome research into effective therapeutic applications. In the context of metabolic diseases, these challenges are amplified because microbial activation of ginsenosides, inter-individual microbiome variability, and inconsistencies in ginseng formulations directly influence metabolic efficacy and reproducibility [15,16,24].

a Inter-individual microbiome variability

One of the most significant barriers is the high degree of interindividual variability in gut microbiota composition, which critically shapes the metabolic conversion of ginsenosides and thereby influences downstream effects on glucose and lipid metabolism. Specifically, variations in the abundance and activity of microbial glycosidases enzymes such as β-glucosidase (commonly associated with the gene *bglX*) and α-L-rhamnosidase (*rhaM*) determine the capacity to generate active metabolites like compound K from precursor ginsenosides [63]. Variability in these gene profiles across individuals leads to heterogeneity in therapeutic responses, even when identical ginseng regimens are administered. Emerging tools such as shotgun metagenomics [64], combined with predictive bioinformatics platforms like PICRUSt [65], offer the potential to characterize baseline microbial function and predict metabolic responsiveness to ginseng based on an individual's enzymatic capacity. These approaches lay the groundwork for personalized microbiome-guided strategies that match ginseng treatment to a person's specific microbial enzymatic capacity.

b Standardization of ginseng preparations

Another critical challenge is the lack of standardization across ginseng products. The type of ginseng (e.g., *Panax ginseng* vs. *Panax quinquefolius*), processing method (e.g., red, white, fermented), dosage, and administration route, all critically influence the chemical profile of ginsenosides and their microbial biotransformation potential [66]. The complexity of ginseng, containing dozens of ginsenosides and polysaccharides, makes it difficult to assign observed effects to attribute specific effects to individual compounds without detailed fractionation and functional testing [67]. Developing standardized preparations, such as well-characterized whole extracts or purified fractions, is critical for ensuring consistent microbial and host responses. Such standardization is especially important for metabolic research, where small differences in ginsenoside composition can lead to substantial variation in lipid or glucose regulation outcomes.

c. Microbiome-focused human clinical trials

A significant gap exists in the design of human clinical trials, as many studies have been underpowered and have not focused on microbiome-specific endpoints, making it challenging to draw robust conclusions regarding the role of the gut microbiota in mediating ginseng's clinical effects. Although 16S rRNA gene sequencing is widely used in clinical studies for taxonomic profiling, it lacks the resolution required to distinguish strains and to provide functional insights into metabolic activity. To overcome these limitations, future trials should integrate advanced multi-omics approaches such as shotgun metagenomics, metabolomics, and host transcriptomics [68]. These technologies will enable researchers to uncover specific microbial pathways and biomarkers predictive of treatment response. This is particularly relevant in high-variability populations, such as elderly individuals or patients with metabolic syndrome, where personalized interventions are most needed [69]. Larger, well-powered randomized controlled trials with clearly defined microbiome and metabolite endpoints are essential for validating clinical efficacy and informing the development of precision biomarkers.

d Advanced mechanistic models

Mechanistic models are essential for elucidating the complex dynamics between ginseng and the gut microbiota. Recent advances in sequencing and computational modeling have enabled deeper insights. Techniques such as the reconstruction of metagenome-assembled genomes (MAGs) and single-cell genomics facilitate the discovery of uncultured microbial species and novel enzymes involved in ginsenoside deglycosylation [70]. Integrating artificial intelligence and machine learning with multi-omics data (e.g., metagenomic, metabolomic, and transcriptomic) further enhances our ability to predict individual responses and guide therapeutic optimization [71]. These approaches have illuminated how ginseng selectively promotes beneficial bacteria such as *Bifidobacterium* and *Akkermansia*, while inhibiting pathogenic microbes. They also clarify how microbial communities mediate ginsenoside transformation and absorption, thereby explaining interindividual variability in therapeutic outcomes [15,16].

Animal models remain indispensable for establishing causality. Gnotobiotic mouse models and FMT studies are gold standards for proving that microbiota changes mediate host responses to ginseng [72]. Other rodent models such as diet-induced obesity or inflammation models also provide useful insights into how ginseng–microbiota interactions influence disease phenotypes. Additionally, simpler model organisms like *Caenorhabditis elegans* or *Drosophila melanogaster* offer technical advantages for high-throughput screening and genetic manipulation. Although these models do not fully recapitulate the complexities of human physiology, these models are highly useful for dissecting microbial metabolite–host signaling pathways. The integration of diverse experimental models with advanced tools is essential for unraveling ginseng–microbiome interactions and accelerating therapeutic applications.

e Regulatory considerations

The clinical translation of ginseng–microbiome interventions faces regulatory challenges. Currently, regulatory agencies lack clear classifications for ginseng-based products, particularly when used alongside probiotics or designed to specifically modulate gut microbiota. The inherent variability in both ginseng composition and individual microbiota profiles complicates standardization efforts and raises questions about safety, efficacy, and labeling. Establishing internationally harmonized quality control measures, manufacturing standards, and definitions for clinical endpoints is crucial to support the regulatory approval of ginseng-based microbiome therapies [73].

To address these challenges, future efforts should focus on methodological innovation, precision medicine approaches, and rigorously designed, well-powered human trials. As multi-omics tools mature and regulatory bodies evolve to incorporate microbiome science, ginseng-based therapies may be positioned as safe, effective, and personalized interventions for a wide range of chronic diseases.

## 6 Conclusion

Mounting evidence demonstrates that the systemic health benefits of ginseng are largely mediated by its dynamic interactions with the gut microbiota. Among these benefits, metabolic regulation emerges as the most consistently supported and mechanistically defined domain, with microbial metabolites acting as key intermediaries linking ginseng intake to improvements in glucose homeostasis, lipid metabolism, and hepatic function. Microbial biotransformation of ginsenosides generates bioactive metabolites such as compound K, which contribute to AMPK activation, enhanced insulin signaling, and attenuation of hepatic steatosis. Ginseng further remodels the gut microbial community, increasing SCFA-producing and bile acid–modulating taxa, thereby enriching microbial metabolites that regulate lipid oxidation, intestinal barrier integrity, and bile acid–FXR/TGR5–AMPK signaling. Mechanistic studies using gnotobiotic animal models, FMT, and multi-omics analyses have provided causal evidence for microbiome-mediated effects of ginseng.

**Fig. 2:**
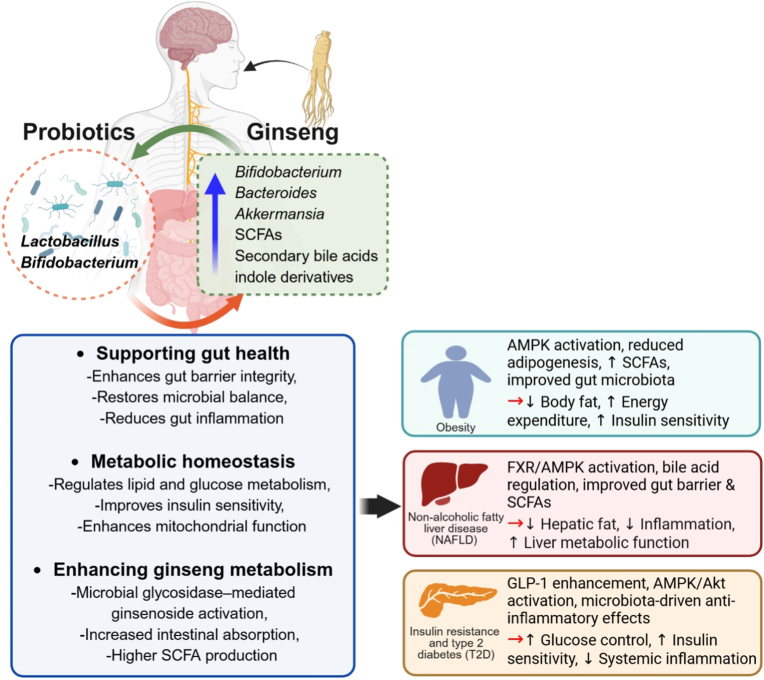
**Microbiota-mediated metabolic benefits of ginseng: therapeutic convergence with probiotics.** Ginseng intake preferentially enriches probiotic taxa, particularly *Bifidobacterium* and *Lactobacillus*, and increases microbial production of short-chain fatty acids (SCFAs), secondary bile acids, and indole derivatives that regulate lipid, glucose, and energy metabolism. These metabolites reinforce epithelial barrier integrity and contribute to systemic metabolic homeostasis by modulating fatty acid oxidation, insulin signaling, and mitochondrial energy pathways. In parallel, probiotic strains encoding glycosidases biotransform ginsenosides into pharmacologically active metabolites, improving bioavailability and systemic exposure. Co-administration of ginseng with such probiotics represents a synergistic metabolic intervention capable of correcting microbial dysbiosis and improving key metabolic features of obesity, insulin resistance, and non-alcoholic fatty liver disease (NAFLD).

Notably, ginseng exhibits mechanistic convergence with probiotic genera such as *Bifidobacterium* and *Lactobacillus*, which can enhance ginsenoside metabolism while also supporting SCFA production, gut barrier integrity, and bile acid signaling. These findings suggest that combining ginseng with probiotic strains capable of both supporting gut health and enhancing ginsenoside metabolism may represent a promising strategy for improving metabolic homeostasis (Fig. 2, Table 1, Table 2).

**Table 2:** Probiotic strains: functions & mechanisms converging with ginseng's effects.

Probiotic strain	Function	Mechanism (converging with ginseng)	Reference
*L*. *plantarum* LMT1-48	Anti-obesity	Reduces body weight, fat, and liver triglycerides by downregulating lipogenic genes (PPARγ, C/EBPα, FAS, FABP4); exhibits antimicrobial activity against *E. cloacae* and inhibits adipocyte differentiation	[[45], [46], [47]]
*L. plantarum* HAC01	Anti-diabetic	Improves glucose metabolism and protects pancreatic β-cells; activates AMPK and inhibits MAPK signaling, reducing adipogenesis and mesenteric fat.	[48,49]
*L. plantarum* SKO-001	Anti-obesity	Lowers body fat and improves lipid metabolism by regulating adipogenesis pathways	[50,51]
*L. plantarum* Q180	Anti-obesity	Inhibits lipid accumulation, adipocyte differentiation, and decreases LDL-cholesterol and apolipoprotein	[52]
*L. plantarum* TWK10	Exercise performance	Enhances mitochondrial function and oxygen utilization, improves grip strength, endurance, and reduces fatigue biomarkers	[53,54]
*B*. *breve* BR03 & B632	Anti-obesity	Improves insulin sensitivity in obese children, supports gut microbiota balance	[55]
*B. lactis* HN019	Gut integrity, immune support	Enhances tight junctions, modulates innate immunity, reduces diarrhea and improves bowel function	[56,57]
*B. breve* B-3	Anti-obesity, gut barrier integrity	Strengthens tight junction proteins, suppresses fat accumulation, and regulates bile acid metabolism; improves cholesterol and glucose levels	[58,59]

PPARγ, peroxisome proliferator-activated receptor gamma; C/EBPα, CCAAT/enhancer-binding protein alpha; FAS, fatty acid synthase; FABP4, fatty acid-binding protein 4; AMPK, AMP-activated protein kinase; MAPK, mitogen-activated protein kinase; LDL, low-density lipoprotein.

Despite this promise, clinical translation remains constrained by variability in microbiota composition and enzymatic capacity and by heterogeneity in ginseng preparations. Future research integrating standardized ginseng formulations, microbiome profiling, and multi-omics–based clinical designs will be essential to advance microbiome-informed ginseng therapies targeting obesity, NAFLD, insulin resistance, and related metabolic disorders.

## CRediT authorship contribution statement

Woo Kyu Kang: Writing – original draft, Formal analysis, Conceptualization, Investigation, Sun-Young Hwang: Writing – review & editing, Revision, Resources, Hyunjin Kang: Writing – review & editing, Jin Won Hyun: Writing – review & editing, Sang-Kyu Kim: Writing – review & editing, Supervision, Funding acquisition, Conceptualization, Mee-Hyun Lee: Writing – review & editing, Supervision, Funding acquisition, Conceptualization, Funding, Administration

## Declaration of competing interest

The authors declare that they have no known competing financial interests or personal relationships that could have appeared to influence the work reported in this paper.

## Data Availability

No data was used for the research described in the article.
